# Weight bias internalization and beliefs about the causes of obesity among the Canadian public

**DOI:** 10.1186/s12889-023-16454-5

**Published:** 2023-08-24

**Authors:** Vida Forouhar, Iyoma Y. Edache, Ximena Ramos Salas, Angela S. Alberga

**Affiliations:** 1 Department of Health, Kinesiology and Applied Physiology, Concordia University, Montreal, Quebec Canada; 2 School of Population and Public Health, University of British Columbia, Vancouver, Canada; 3 Replica Communications, Kristianstad, Sweden; 4 Department of Pediatrics, Faculty of Medicine, McGill University, Montreal, Quebec Canada

**Keywords:** Weight bias, Stigma, Internalized attitudes, Obesity, Public health, Public beliefs

## Abstract

**Background:**

Explicit weight bias is known as negative attitudes and beliefs toward individuals due to their weight status and can be perpetuated through misconceptions about the causes of obesity. Individuals may also experience weight bias internalization (WBI) when they internalize negative weight-related attitudes and self-stigmatize. There is a paucity of research on the beliefs about the causes of obesity and the prevalence of WBI among public Canadian samples. The aim of this study was to describe these attitudes and beliefs about obesity among a large Canadian sample across the weight spectrum.

**Methods:**

A Canadian sample of adults (*N* = 942; 51% Women; mean age group = 45–54 years; mean body mass index [BMI] = 27.3 ± 6.7 kg/m^2^) completed an online questionnaire. Participants completed the Modified Weight Bias Internalization Scale, the Anti-Fat Attitudes Questionnaire, and the Causes of Obesity Questionnaire.

**Results:**

Mean WBI score within the entire sample was 3.38 ​​ ± 1.58, and females had higher mean scores as compared to males (*p* < 0.001). Mean scores were also higher among individuals with a BMI of > 30 kg/m^2^ (4.16 ± 1.52), as compared to individuals with a BMI of 25–30 kg/m^2^ (3.40 ± 1.50), and those with a BMI of 20–25 kg/m^2^ or below 18.5 kg/m^2^ (2.81 ± 1.44) (*p* < 0.001 for all). Forty four percent of Canadians believed behavioural causes are very or extremely important in causing obesity, 38% for environmental causes, 28% for physiological and 27% for psychosocial causes. Stronger beliefs in behavioural causes were associated with higher levels of explicit weight bias. No BMI differences were reported on the four different subscales of the Causes of Obesity Questionnaire.

**Conclusions:**

Weight bias internalization is prevalent among Canadians across all body weight statuses, and the public endorses behavioural causes of obesity, namely physical inactivity and overeating, more than its other causes. Findings warrant the reinforcement of efforts aimed at mitigating weight bias by educating the public about the complexity of obesity and by highlighting weight bias as a systemic issue that affects all Canadians living in diverse body weight statuses.

## Introduction

Weight bias is a social justice concern in Canada [1], where 61% of Canadian adults who participated in commercial weight management programs reported experiences of weight-based discrimination throughout their lives [2]. Explicit weight bias is defined as negative attitudes and beliefs about individuals due to their weight status [3] and affects the lives of individuals perceived as having a higher weight on a daily basis, be it in the workplace, in education settings, in the media, or even in healthcare settings [4, 5]. This form of bias is related to the socially acceptable stereotypes that individuals in larger bodies, particularly individuals with overweight and obesity, are lazy, incompetent, and lack willpower [6, 7]. Weight bias also derives from societal misconceptions surrounding the causal attributions of obesity (beliefs about the causes of obesity) [8], namely that obesity is mainly attributed to behavioural factors that are solely within individual control, such as physical inactivity [9]. Individuals may also internalize these negative societal beliefs and apply it to themselves at the detriment of their own self-esteem [10]. This self-directed form of weight bias, known as weight bias internalization (WBI), affects not only individuals with obesity but all individuals across the weight spectrum [11, 12]. While the experiences of weight bias have been well-documented among treatment-seeking samples, as seen in a multinational study by Puhl and colleagues [2], there has been less focus on WBI and beliefs about the causes of obesity among public samples of people across the weight spectrum.

In Canada, only one study assessed the prevalence of WBI among a convenience sample of adults and found that participants internalize weight bias to some extent, with higher levels among women as compared to men [13]. Similarly, only one study measured beliefs about the causes of obesity among Canadians [14], but only focused on its association with explicit weight bias, rather than on reporting how individuals attribute obesity to various factors (i.e. behavioural, environmental, etc.). Other studies from a systematic review reporting on American and German public beliefs found that the most frequently endorsed factors that contribute to obesity were behavioural factors, including physical inactivity and dietary patterns [9]. It is important to note that these beliefs may be potentially related to weight bias, whether explicit or internalized [9, 15, 16]. For instance, in a large multinational study measuring weight bias across four countries including Canada, the United States, Iceland, and Australia, stronger beliefs in behavioural factors of obesity were associated with more negative attitudes towards individuals with obesity (explicit weight bias)[14]. Further, findings suggested potential BMI differences, as individuals with obesity in the Canadian and Icelandic samples demonstrated lower levels of weight bias, which should be further explored in order to understand who to target with improved education on the complexity of obesity and weight bias reduction programs.

To date, studies on WBI have reported these attitudes mainly among women with overweight and obesity, and studies reporting on the beliefs about the causes of obesity have used samples with experts on obesity; no study has examined these two constructs together in a large public Canadian sample. Given that negative attitudes about obesity, weight-related self-stigma, and misconceptions about the factors related to obesity can perpetuate weight bias and contribute to weight discrimination (i.e. the unfair treatment of individuals because of their weight), it is important to understand the prevalence of these beliefs among Canadians in order to better address weight bias as a systemic issue. These data could help to bring awareness to this social justice issue, to inform public anti-discrimination policies and to design future weight bias reduction interventions through obesity education and more upstream public health initiatives.

The primary objectives of the present study were to: (1) assess the prevalence of internalized weight bias among the Canadian public, and (2) to describe how Canadians attribute obesity to different causes. The secondary objectives of this study were to assess whether there are differences in mean WBI scores between males and females and according to BMI, and if beliefs about the causes of obesity differ according to BMI. Further, the relationship between explicit weight bias and the different causes of obesity was assessed. It is hypothesized that Canadians will demonstrate weight bias internalization to some extent and will attribute obesity mainly to behavioural causes. It is also hypothesized that females and individuals with higher BMIs will have higher levels of weight bias internalization and that behavioural causes of obesity will be most endorsed by individuals with lower BMIs. Those with higher levels of weight bias will endorse more behavioural factors compared to other factors.

## Method

### Participants and Procedure

A secondary analysis was conducted on previously collected data as part of the cross-sectional study, *Canadian Public Support for Obesity Public Policies* [17]. Potential participants were recruited using an online market research company, known as Survey Sampling International (SSI), in order to generate a representative sample of English-speaking Canadian adults over the age of 18. To allow for an approximation of Canadian demographics, quotas based on age, sex, and province of residence were gathered by SSI. Representativeness was determined once recruitment matched the predetermined demographic quotas. Prior to official recruitment, a total of 42,080 eligible participants were invited to complete a survey and received emails describing the study purpose, length of the survey, and compensation for participation. SSI used an array of fraud detection tools in order to identify and filter out fraudulent responses. Strategies such as digital fingerprinting, tests for logical answer consistency, and measurements of response time were implemented while respondents completed the surveys through the SSI platforms. Initial interest in participation was expressed by 1865 participants who clicked on the survey, and a total of 1588 participants submitted survey responses (response rate: 3.7%). Individuals who did not complete the full survey were removed, leaving a total of 942 participants (completion rate: 59%). Even though 646 participants were removed for various reasons (not completing the survey beyond demographics, missingness, and inconclusive demographic data), the demographic characteristics of the final sample of 942 participants was still relatively comparable to the demographic quotas that were used to generate the initially recruited sample of 1588 participants. All participants who volunteered to take part in this study completed an informed consent form prior to participation. Ethics approval for all aspects of this study was granted by a Research Ethics Board (Ethics certificate number: 30009752).

### Measures

#### Demographic Variables

The demographics section of this questionnaire consisted of questions assessing age, sex, ethnicity, and self-reported measures of height and weight to calculate BMI.

#### Weight Bias Internalization

The Modified Weight Bias Internalization Scale (WBIS-M) was used in order to assess the extent to which individuals internalize negative attitudes about weight [18–20]. The 11 items on this validated questionnaire were rated using a 7-point Likert scale (1 = strongly disagree, 7 = strongly agree), with higher scores indicating higher WBI. Items 1 and 9 reflect positive attitudes about weight, therefore these items were reversed scored. An example of an item from this questionnaire is “I feel anxious about my weight because of what people might think of me”. In two distinct studies assessing psychometric properties of this questionnaire, the first item: “Because of my weight, I feel that I am just as competent as anyone” did not show adequate internal consistency, and once it was removed from the analysis, the overall internal consistency was improved. For the purpose of this study, internal consistency was assessed with and without the first item, and the results concurred with these previous studies. The overall internal consistency improved from a Cronbach’s alpha score of 0.92 to 0.94, thus the first item was removed from further analyses.

#### Causes of Obesity

The Causes of Obesity Questionnaire (COB) was used in order to assess beliefs about the different causes of obesity. This validated questionnaire consists of 14 items that responders have to rate in terms of how important they believe they are in causing obesity. Items on the COB are assessed on a 5-point Likert scale (1 = not at all important, 5 = extremely important) [20, 21]. Given that this questionnaire does not have any predetermined subscales, for this study the 14 different items were divided into subscales based on the results of an exploratory factor analysis [14]. The four main subscales derived from this factor analysis were behavioural causes (e.g. physical inactivity), environmental causes (e.g. pricing of foods), physiological causes (e.g. metabolic disorder), and psychosocial causes (e.g. psychological problems). In this study, the COB demonstrated good-to-strong internal consistency with Cronbach’s alpha scores for the behavioural, environmental, physiological and psychosocial causes of 0.80, 0.70, 0.83, and 0.74 respectively, and 0.88 for the entire scale.

#### Explicit Weight Bias

The Anti-Fat Attitudes Questionnaire (AFA) was used to assess explicit weight bias. This validated questionnaire contains 13 items separated into three subscales that represent the three main domains of explicit anti-fat attitudes: *Dislike* (*n* = 7 items), *Fear of Fat* (*n* = 3 items), and *Willpower *(*n* = 3 items) [20, 22]. The *Dislike* subscale assessed negative attitudes toward individuals with obesity, (e.g., “I really don’t like obese people much”). The *Fear of Fat* subscale assessed an individual’s fear of gaining weight (e.g., “I feel disgusted with myself when I gain weight”). The *Willpower* subscale assessed perceptions that weight gain or obesity is within individual control (e.g., “Some people are obese because they have no willpower”). All of the items in each subscale are rated on a 10-point Likert scale (0 = very strongly disagree, 9 = very strongly agree). A total score above zero represents the presence of weight bias, with higher scores indicating greater weight bias or more anti-fat attitudes. In this study, the AFA demonstrated strong internal consistency with Cronbach’s alpha scores for the *Dislike*, *Fear of Fat*, and *Willpower* subscales of 0.88, 0.85, and 0.81 respectively, and 0.87 for the entire scale.

### Data analysis

All statistical analyses were conducted using R and JASP. Descriptive statistics were used to describe the data as means and standard deviations for weight bias internalization and beliefs about the causes of obesity. An exploratory factor analysis was conducted to separate the items on the Causes of Obesity Questionnaire (COB) into different subscales. The 14 different items on the questionnaire were separated into the following four subscales: behavioural causes, environmental causes, physiological causes, and psychosocial causes. This test was done as a preliminary and necessary step to conduct the following analyses on the COB, as the aim was to describe the data based on the four overarching subscales found [14]. Frequency tables were used to compare the percentage of endorsement on each separate cause of obesity, as well as on the four separate subscales, and a MANOVA was run to assess BMI differences. Mean WBI scores were analyzed for differences among men and women using an independent t-test, as well as for BMI differences using a one-way between measures ANOVA. A correlational analysis was done to determine the relationship between BMI and WBI. Participants who scored 1 standard deviation (SD) above the mean WBI were categorized as having “high” WBI, those who scored within 1 SD of the mean were categorized as “average” and those who scored 1SD below the mean were categorized as having “low” WBI. A linear regression was conducted to determine the relationship between mean scores on the Anti-Fat Attitudes Questionnaire and the four different factors of the Causes of Obesity Questionnaire. The regression was adjusted for age, sex, ethnicity and BMI.

For the purpose of analyses in this paper, participants’ BMIs were calculated using their self-reported measures of height and weight and were classified into different groups according to the guidelines from Health Canada: underweight (BMI < 18.5 kg/m^2^), normal weight (BMI = 18.5–24.9 kg/m^2^), overweight (BMI = 25.0–29.9 kg/m^2^), and obesity (BMI > 30.0 kg/m^2^) [23]. Few participants were originally classified as living with underweight (*n* = 36) and were therefore grouped into the normal weight category for analyses, in order to have a more even distribution between BMI groups. The three final BMI groups for analyses were normal and underweight, overweight, and obesity.

## Results

### Descriptive characteristics

The study’s sample characteristics are described in Table 1. A total of 942 participants were included in the final sample. The demographic characteristics (sex, age, and race/ethnicity) of the final study sample were relatively comparable to that of the Canadian population (comparisons made with 2016 Canadian Census data) [24]. Fifty one percent of participants in the sample were female (*n* = 484), while 48% were male (*n* = 450), and a few participants identified as “other”, at 0.85% (*n* = 8). The average age range of the sample was 45–54 years, and average BMI was 27.3 ± 7 kg/m^2^. The majority of the sample was White (74.3%), followed by Asian (10.6%), South Asian (3.0%), Black/African/Caribbean (2.9%), Aboriginal Peoples (2.6%), Other (2%), Middle Eastern (1.3%), Southeast Asian (1.3%), Hispanic/Latin American (1.1%), Biracial/Biethnic (0.9%), and Pacific Islander (0.2%). Given that there were few participants who identified as "other" (0.85%), a binary sex comparison was made between males and females only. Data from these individuals were included in all other analyses.

**Table 1:** Sample Characteristics

Measure	Total Sample (*N* = 942)	
Age	***n***	**%**
18–24	106	11.2
25–34	171	18.1
35–44	168	17.8
45–54	204	21.6
55–64	169	17.9
65 +	124	13.2
Sex		
Male	450	47.8
Female	484	51.4
Other	8	0.85
Body Mass Index		
Underweight	36	3.8
Normal Weight	334	35.5
Overweight	311	33.0
Obesity	261	27.7
Race/Ethnicity		
White	700	74.3
Non-White	242	25.7
Asian	100	10.6
South Asian	28	3.0
Black/African/Caribbean	27	2.9
Aboriginal	24	2.6
Other	19	2.0
Middle Eastern	12	1.3
Southeast Asian	12	1.3
Hispanic/Latin American	10	1.1
Biracial/Bi-Ethnic	8	0.9
Pacific Islander	2	0.2
	**M**	**SD**
Weight Bias Internalization Scores		
Total Sample	3.38	1.58
Males	3.16	1.48
Females	3.58	1.65
Normal Weight & Underweight	2.81	1.44
Overweight	3.40	1.50
Obesity	4.16	1.52
Causes of Obesity Subscale Scores		
Behavioural Causes	3.70	0.82
Psychosocial Causes	3.36	0.82
Environmental Causes	3.34	1.03
Physiological Causes	3.28	0.92

### Weight bias internalization

**Fig. 1:**
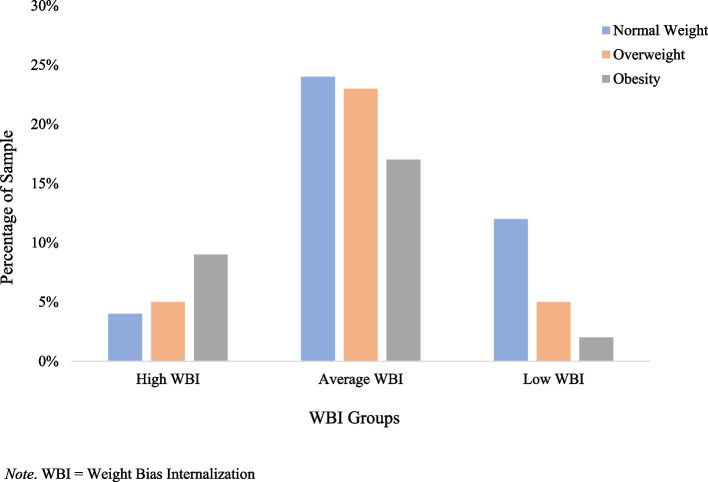
Mean Weight Bias Internalization by Body Mass Index Distribution

Within our entire sample, the mean WBI score was 3.38 ​​ ± 1.58, with significantly higher mean scores among females (3.58 ± 1.65) as compared to males (3.16 ± 1.48, *p* < 0.001). The majority of the sample fell within the mean range for WBI at 63%, with 19% of participants categorized as having “low” WBI, and 18% as having “high” WBI. To assess BMI differences on mean WBI scores, participants were classified into three different groups: normal weight and underweight (*n* = 370), overweight (*n* = 311), and obesity (*n* = 261). Among the participants who had high WBI scores, 51% were classified in the obesity BMI group, 29% were individuals classified in the overweight BMI group, and 20% were classified in the normal weight and underweight BMI group. A depiction of high WBI scores by BMI group can be seen in Fig. 1. Mean scores were statistically significantly different between BMI groups, *F* (2, 940) = 125.9, *p* < 0.001, η^2 = 0.12. WBI scores were lower for the normal weight and underweight group (*M* = 2.81, *SD* = 1.44) as compared to the overweight group (*M* = 3.40, *SD* = 1.50), and the obesity group had the highest WBI scores (*M* = 4.16, *SD* = 1.52). Post hoc analysis with a Bonferroni correction showed that the difference in mean scores between the normal/underweight group and overweight group, the normal/underweight group and obesity group, and between the overweight group and obesity group, were all statistically significant (*p* < 0.001 for all).

### Beliefs about the causes of obesity

**Fig. 2:**
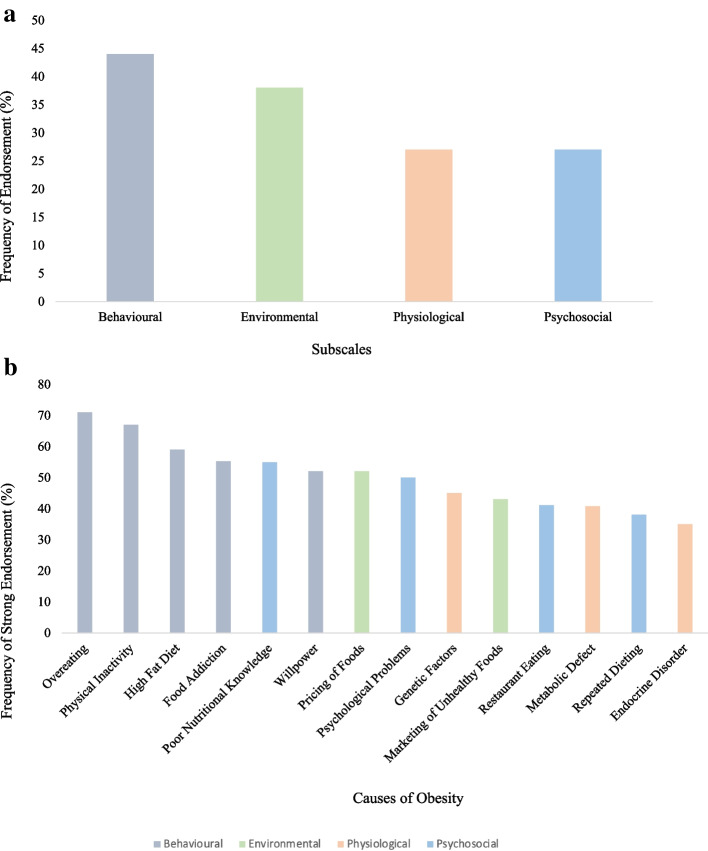
**a** Frequency of Endorsement of the Causes of Obesity by Subscale. **b** Frequency of Endorsement of the 14 Causes

Canadians attribute obesity mainly to behavioural causes as compared to other causes. Forty four percent of the sample believed that behavioural factors are very or extremely important in causing obesity, compared to 38% for environmental causes, and only 28% and 27% for physiological and psychosocial causes, respectively. Among the most endorsed causes of obesity were overeating, physical inactivity, and a high fat diet, at 71%, 67%, and 59% of the sample, respectively. Among the least endorsed causes were endocrine disorders, repeated dieting, and metabolic factors, at 35%, 38%, and 41% of the sample, respectively. An ANOVA revealed no statistical differences between the mean scores of the four subscales according to BMI categories. Beliefs in behavioural causes of obesity were positively associated with explicit weight bias scores (*B* = 0.46, *t*(921) = 5.58, *p* < 0.001), while beliefs in physiological causes of obesity were negatively associated with explicit weight bias scores (*B* = -0.16, *t*(921) = -2.14, *p* < 0.05). Explicit weight bias was not associated with beliefs in psychosocial or environmental causes of obesity (*p* = 0.50, *p* = 0.37). All these results are shown in Figs. 2a and 2b, and Table 2. The *Lack of Willpower* item on the Causes of Obesity Questionnaire was weakly correlated with the *Dislike* and *Fear of Fat subscales*, but moderately correlated with the *Willpower* subscale of the Anti-Fat Attitudes Questionnaire (r = 0.11, r = 0.21, r = 0.42; respectively)

**Table 2:** Linear Regression: Explicit Weight Bias and Beliefs about the Causes of Obesity

Variable	**Explicit Weight Bias (*B*) (*SE*)**
Sample (*N* = 921)	
COB Subscale mean scores	
Behavioural Causes	0.46 (0.82)***
Physiological Causes	-0.16 (0.73)*
Psychosocial Causes	0.06 (0.95)
Environmental Causes	0.56 (0.06)

*Note. B* Parameter estimate, *COB* Causes of Obesity. * *p* < .05. ** *p* < .01. *** *p* < .001. **** *p* < .0001 Adjusted for age, sex, ethnicity and body mass index (BMI)

## Discussion

Results from this study showed that Canadians demonstrated WBI to some extent and endorsed behavioural causes of obesity more than physiological, psychosocial and environmental causes. Females and individuals with higher BMIs had higher mean WBI scores, however a considerable portion of the sample who were categorized under the normal weight and underweight BMI category also expressed high levels of WBI. Beliefs in behavioural causes of obesity were associated with more explicit weight bias, while beliefs in physiological causes of obesity were associated with less explicit weight bias. The endorsement of the different causes of obesity did not differ by BMI.

Our study adds to the literature by providing comparable results to the few studies that measured WBI in population-based samples. The mean WBI of 3.38 in our study sample is comparable to the mean WBI in a sample of 2529 American adults (*M* = 3.36) generated from SSI, and relatively comparable to another sample from the same study of 519 American adults (*M* = 3.31) generated from an online data source called Mechanical Turk [25]. These mean scores are lower in comparison to samples of individuals who reported struggling with their weight, treatment seeking adults with obesity, and individuals considering bariatric surgery (*M* = 4.72, *M* = 4.60, and *M* = 4.54, respectively); an expected result given the association between BMI and WBI. Only 18% of the participants in the study sample scored within the “high” WBI category, relative to the sample mean. Among these participants, 20% were individuals with a BMI in the normal weight and underweight category. Although these results are only relative within our sample mean, they demonstrate the persistence of WBI, even at high levels, among those who have a BMI in the normal or underweight range. High WBI was also found among individuals with higher BMIs, consistent with previous research that has documented an association between higher internalized weight bias levels and high BMIs [19, 26–28]. Individuals living with overweight and obesity are subjected to weight bias, stigmatization and discrimination and are therefore more susceptible to internalizing negative attitudes about weight [19, 26]. Results from the Canadian ACTION study showed that individuals with obesity believe that obesity management falls under the responsibility of the individual [29], which may be reflective of internalizing weight bias. Although there are BMI differences in weight bias internalization, it does not dispute the finding that individuals with normal weight and underweight also experience high WBI. This is important to note in efforts to change the narrative around obesity, as it alleviates the misconception that only individuals with higher body weight are affected by the perils of weight bias, and further highlights this as a larger systemic issue affecting the wellbeing of all individuals regardless of weight or size.

Levels of WBI differed according to sex with higher mean scores among females as compared to males. This finding is consistent with studies in the literature measuring WBI among samples of adults with overweight and obesity [11, 19, 27, 30] as well as in one sample with Canadian adults of all body weight statuses [13]. However, direct comparisons are difficult to make as these previous studies reported gender differences between men and women and not sex differences. Due to societal pressures to conform to beauty standards, women are typically more vulnerable to biases based on physical appearance [14, 26]. The idealization of, and drive for, attaining a “thinner” body among women may contribute to this internalization of negative attitudes toward weight and weight gain [19]. Women also express more body weight and shape concerns than men, which can play a role in weight bias internalization [27, 28, 31]. The majority of the studies assessing this relationship found no gender differences in this relationship, however most samples consisted of mainly women with overweight and obesity [19, 28, 31]. Future research would benefit from accurately capturing sex and exploring sex differences in WBI according to levels of body image and body weight concerns, among representative samples of the general population. Future studies should also be inclusive of individuals with diverse gender identities and appropriately capture gender to determine these differences in BMI.

There were no differences in BMI between the four Causes of Obesity subscales, this may be due to an engrained societal belief, among individuals of all body weight statuses, that obesity is primarily a behavioural problem that is caused by an inability to have control over one’s weight. This finding may also be linked to the level of weight bias internalization, as individuals who internalize negative attitudes about their own weight might also endorse the belief that obesity is primarily attributed to behavioural problems. Future research should explore whether WBI plays a role in the association between BMI and beliefs about the causes of obesity.

Consistent with our hypotheses and in line with results from a systematic review, our results showed that more Canadians believed behavioural causes of obesity were very or extremely important in causing obesity as compared to environmental, physiological, and psychosocial causes. In one of the studies in this systematic review [32], as well as in a multinational study assessing weight bias across Canada, Iceland, Australia and the United States [14], beliefs in behavioural causes of obesity were associated with higher explicit weight bias; comparable to the results found in our study. Attributing obesity to behavioural factors lies within the belief that obesity is a condition within individual control, which reinforces negative stereotypes about these individuals and perpetuates weight bias. We can speculate from these results that educating the public on the complexity of obesity as a chronic disease may help to reduce these negative weight-related attitudes. These results could inform future interventions aiming to determine if education on obesity can impact weight-bias attitudes, whether toward others or self-directed through WBI. The results from this study could inform knowledge translation outputs to first disseminate information about the prevalence of weight bias among Canadians, and to also take action on reducing weight bias among the public through advocacy initiatives.

To the best of our knowledge, this is the first study to describe beliefs about the causes of obesity and levels of weight bias internalization among a sample of Canadian adults taken from the general population, inclusive of individuals across the weight spectrum. Our study had an equal representation of males and females, unlike other studies on weight bias internalization, which had samples of predominantly females or women living with overweight and obesity. This study was also the first to assess beliefs about the causes of obesity using a validated questionnaire. However, the findings in this study should be interpreted within the context of its limitations. Given the nature of a cross-sectional study, no causal relationships can be deduced, and no follow-up data was generated to assess changes in attitudes overtime; future longitudinal research is warranted to determine how these attitudes evolve. There were also no objective measures of any of the variables measured, as self-reported questionnaires are susceptible to social desirability bias and inaccuracies, particularly for height and weight measurements. Research shows that individuals tend to under-report their weight and over-report their height, which could have affected the BMI data reported in this study [33]. Moreover, there is no way of knowing if the participants in this study were given a clinical obesity diagnosis, since BMI was used a proxy measure to reflect obesity. Another limitation of this study was that other we did not have a measure of gender identity that is inclusive of all underrepresented groups. For this reason, it was not possible to conduct gender analyses and only binary sex analyses between males and females were conducted. This sample also had an overrepresentation of individuals who identified as white, future research should be more inclusive of individuals from other ethnic groups. Although our sample consisted of individuals across the weight spectrum, participants categorized into the underweight BMI group were underrepresented, and therefore grouped with those in the normal weight group for analyses. Future research should compare outcomes between individuals categorized in the underweight BMI group and those categorized in the normal weight BMI group as there may be important differences to note between the outcomes of these two groups.

Additionally, results on the beliefs about the causes of obesity might be biased, as the Causes of Obesity Questionnaire (COB) itself has a larger representation of behavioural causes of obesity (*n* = 5 items) as compared to psychological (*n* = 4 items), physiological (*n* = 3 items), and environmental causes (*n* = 2 items). This is not ideal for measuring these beliefs as there are multiple causes of obesity from all of these subscales and having an unequal representation of the factors could bias the results toward the most represented subscale, in this case for behavioural causes. This calls for future research to develop new scales that improve upon the limitations of the COB, in order to account for more factors that reflect other causes of obesity like environmental, psychosocial, and physiological causes. These new scales should also demonstrate the multifactorial and complex nature of obesity by considering that obesity is caused by an interaction of these subscales rather than singling them out. Another important limitation of this study is that experiences of weight bias were not measured within the sample. As experiences with weight bias have been linked to weight bias internalization, including this information would have allowed for a greater understanding of how these measures of weight bias interrelate among a general population sample of adults in Canada.

## Conclusion

Our study provides a comprehensive overview of public attitudes on the various causes of obesity as well as insight into levels of weight bias internalization among a large sample of Canadian adults. Weight bias internalization is prevalent among Canadians across all body weight statuses, with higher levels among females as compared to males. Moreover, the Canadian public endorses behavioural causes of obesity more than environmental, physiological, and psychosocial causes, which is related to more negative attitudes and bias against individuals with obesity. Results from this study may urge policy makers to educate the public on the complex causes of obesity beyond behavioural factors and push forward the agenda of changing the narrative around obesity, particularly around weight bias, as it is a social justice concern that extends beyond just individuals with obesity.

## Data Availability

The datasets used and/or analysed during the current study are available from the corresponding author on reasonable request.

## References

[1] Nutter S, Russell-Mayhew S, Arthur N, Ellard JH (2018). Weight bias as a social justice issue: A call for dialogue. Can Psychol.

[2] Puhl RM, Lessard LM, Pearl RL, Himmelstein MS, Foster GD (2021). International comparisons of weight stigma: addressing a void in the field. Int J Obes.

[3] Puhl R, Brownell KD (2001). Bias, Discrimination, and Obesity. Obes Res.

[4] Godley J (2018). Everyday Discrimination in Canada: Prevalence and Patterns. Can J Soc.

[5] Puhl RM, Latner JD, O’Brien KS, Luedicke J, Danielsdottir S, Salas XR (2015). Potential Policies and Laws to Prohibit Weight Discrimination: Public Views from 4 Countries: Potential Policies and Laws to Prohibit Weight Discrimination. Milbank Q.

[6] Puhl RM, Heuer CA (2009). The Stigma of Obesity: A Review and Update. Obesity.

[7] Puhl RM, Heuer CA (2010). Obesity Stigma: Important Considerations for Public Health. Am J Public Health.

[8] Rubino F, Puhl RM, Cummings DE, Eckel RH, Ryan DH, Mechanick JI, Nadglowski J, Ramos Salas X, Schauer PR, Twenefour D, Apovian CM, Aronne LJ, Batterham RL, Berthoud HR, Boza C, Busetto L, Dicker D, De Groot M, Eisenberg D, Flint SW, Huang TT, Kaplan LM, Kirwan JP, Korner J, Kyle TK, Laferrère B, le Roux CW, McIver L, Mingrone G, Nece P, Reid TJ, Rogers AM, Rosenbaum M, Seeley RJ, Torres AJ, Dixon JB (2020). Joint international consensus statement for ending stigma of obesity. Nat Med.

[9] Sikorski C, Luppa M, Kaiser M, Glaesmer H, Schomerus G, König HH, Riedel-Heller SG (2011). The stigma of obesity in the general public and its implications for public health - a systematic review. BMC Public Health.

[10] Pearl RL, Puhl RM, Dovidio JF (2015). Differential effects of weight bias experiences and internalization on exercise among women with overweight and obesity. J Health Psychol.

[11] Schvey NA, White MA (2015). The internalization of weight bias is associated with severe eating pathology among lean individuals. Eat Behav.

[12] Kahan S, Puhl RM (2017). The damaging effects of weight bias internalization. Obesity.

[13] Levy M, Nguyen A, Kakinami L, Alberga AS. Weight bias internalization: Relationships with mental health, physical activity, and sedentary behavior. Stigma and Health. 2021 Sep 2 [cited 2022 Apr 21]; Available from: http://doi.apa.org/getdoi.cfm?doi=10.1037/sah0000336

[14] Puhl RM, Latner JD, O’Brien K, Luedicke J, Danielsdottir S, Forhan M (2015). A multinational examination of weight bias: predictors of anti-fat attitudes across four countries. Int J Obes.

[15] von dem Knesebeck O, Lüdecke D, Luck-Sikorski C, Kim TJ (2019). Public beliefs about causes of obesity in the USA and in Germany. Int J Public Health.

[16] Reinka MA, Quinn DM, Puhl RM (2021). Examining the relationship between weight controllability beliefs and eating behaviors: The role of internalized weight stigma and BMI. Appetite.

[17] Edache IY, Kakinami L, Alberga AS. Weight bias and support of public health policies. Can J Public Health. 2021 May 14 [cited 2021 Jun 1]; Available from: https://link.springer.com/10.17269/s41997-020-00471-7 10.17269/s41997-020-00471-7 PMC8225739 33990876

[18] Durso LE, Latner JD (2008). Understanding Self-directed Stigma: Development of the Weight Bias Internalization Scale. Obesity.

[19] Pearl RL, Puhl RM (2014). Measuring internalized weight attitudes across body weight categories: validation of the modified weight bias internalization scale. Body Image.

[20] Lacroix E, Alberga A, Russell-Mathew S, McLaren L. Weight Bias: A Systematic Review of Characteristics and Psychometric Properties of Self-Report Questionnaires. 2017;15. 10.1159/000475716 PMC5644934 28601888

[21] Foster GD, Wadden TA, Makris AP, Davidson D, Sanderson RS, Allison DB, Kessler A (2003). Primary Care Physicians’ Attitudes about Obesity and Its Treatment. Obes Res.

[22] Crandall CS (1994). Prejudice against fat people: Ideology and self-interest. J Pers Soc Psychol.

[23] Losing Weight, Body Mass Iindex. [cited 2022 Apr 21]. Available from: https://www.nhlbi.nih.gov/health/educational/lose_wt/BMI/bmi_dis

[24] Government of Canada SC. Census Profile, 2016 Census - Canada [Country] and Canada [Country]. 2017 [cited 2023 Jul 14]. Available from: https://www12.statcan.gc.ca/census-recensement/2016/dp-pd/prof/details/page.cfm?Lang=E&Geo1=PR&Code1=01&Geo2=&Code2=&SearchText=Canada&SearchType=Begins&SearchPR=01&B1=All&TABID=1&type=0

[25] Puhl RM, Himmelstein MS, Quinn DM (2018). Internalizing Weight Stigma: Prevalence and Sociodemographic Considerations in US Adults. Obesity.

[26] Pearl RL, Puhl RM (2018). Weight bias internalization and health: a systematic review. Obes Rev.

[27] Boswell RG, White MA (2015). Gender differences in weight bias internalisation and eating pathology in overweight individuals. Advances in Eating Disorders.

[28] Durso LE, Latner JD, Ciao AC (2016). Weight bias internalization in treatment-seeking overweight adults: Psychometric validation and associations with self-esteem, body image, and mood symptoms. Eat Behav.

[29] Sharma AM, Bélanger A, Carson V, Krah J, Langlois M, Lawlor D, Lepage S, Liu A, Macklin DA, MacKay N, Pakseresht A, Pedersen SD, Ramos Salas X, Vallis M. Perceptions of barriers to effective obesity management in Canada: Results from the ACTION study. Clin Obes. 2019;9(5). Available from: https://onlinelibrary.wiley.com/doi/abs/10.1111/cob.12329 10.1111/cob.12329 PMC6771494 31294535

[30] Hayward LE, Vartanian LR, Pinkus RT (2018). Weight Stigma Predicts Poorer Psychological Well-Being Through Internalized Weight Bias and Maladaptive Coping Responses. Obesity.

[31] Pearl RL, White MA, Grilo CM (2014). Overvaluation of shape and weight as a mediator between self-esteem and weight bias internalization among patients with binge eating disorder. Eat Behav.

[32] Hilbert A, Rief W, Braehler E (2008). Stigmatizing Attitudes Toward Obesity in a Representative Population-based Sample. Obesity.

[33] Christian NJ, King WC, Yanovski SZ, Courcoulas AP, Belle SH (2013). Validity of Self-reported Weights Following Bariatric Surgery. JAMA.

